# Fabrication and characterization of zein/carboxymethyl chitosan nanoparticles for co-encapsulation of curcumin and resveratrol

**DOI:** 10.3389/fnut.2025.1641620

**Published:** 2025-09-05

**Authors:** Pan Wang, Ying Yin, Yue Zhang, Jing Shao

**Affiliations:** ^1^Collaborative Innovation Center for Molecular Imaging of Precision Medicine, Shanxi Medical University, Taiyuan, China; ^2^Shanxi Key Laboratory of Molecular Imaging, Shanxi Medical University, Taiyuan, China; ^3^College of Life Science and Technology, Inner Mongolia Normal University, Hohhot, Inner Mongolia, China; ^4^Key Laboratory of Biodiversity Conservation and Sustainable Utilization in Mongolian Plateau for College and University of Inner Mongolia Autonomous Region, Hohhot, China; ^5^Faculty of Health Sciences, University of Macau, Macau, Macao SAR, China; ^6^Key Laboratory of Systems Bioengineering (Ministry of Education), Tianjin University, Tianjin, China

**Keywords:** co-delivery system, curcumin, resveratrol, zein/carboxymethyl chitosan nanoparticles, stability

## Abstract

**Introduction:**

Curcumin and resveratrol are promising nutraceuticals, but their application in functional foods is limited by poor water solubility, low bioavailability, and chemical instability. This study aimed to develop a novel co-delivery system to overcome these challenges.

**Methods:**

Curcumin/resveratrol-zein-carboxymethyl chitosan (CRZC) nanocomplexes were fabricated using an antisolvent precipitation method. The mass ratio of zein to CMCS was optimized. The nanoparticles were characterized for size, encapsulation efficiency (EE), and antioxidant activity. Their stability during storage and simulated gastrointestinal digestion was also evaluated.

**Results:**

The optimized CRZC nanoparticles (zein:CMCS mass ratio of 2:1) exhibited a small particle size (201.6 nm), high encapsulation efficiency (72.90% for curcumin and 78.23% for resveratrol), and superior radical scavenging capacity. Structural characterization confirmed that hydrophobic interactions, electrostatic attraction, and hydrogen bonding were the main forces maintaining nanocomplex integrity. The CMCS coating significantly improved the storage and digestion stability of both encapsulated bioactives.

**Discussion:**

These results demonstrate that the zein-CMCS-based nanocomplex is an efficient and robust delivery system. The CRZC nanoparticles show great potential for the co-delivery of synergistic nutraceuticals to enhance the functionality and application of bioactive compounds in the food industry.

## 1 Introduction

The innovative development of functional foods has emerged as a pivotal research direction in modern nutritional science, primarily aiming to achieve the dual benefits of nutritional fortification and disease prevention through targeted delivery of bioactive components (1–3). Curcumin, a natural diketone polyphenolic compound extracted from turmeric rhizomes, demonstrates significant application potential in functional foods, pharmaceutical formulations, and cosmetic products due to its broad-spectrum antibacterial properties, potent antioxidant capacity, and anti-inflammatory characteristics (4–7). Similarly, resveratrol, a plant-derived stilbene compound found in grapes and other botanical sources, exhibits remarkable antioxidant and neuroprotective functions, has demonstrated anti-obesity and anti-inflammatory effects through modulation of signaling pathways such as NF-κB (8, 9). Notably, both curcumin and resveratrol have been reported to play crucial roles in alleviating ulcerative colitis symptoms and maintaining intestinal function, primarily attributed to their antioxidant and anti-inflammatory activities (10). Studies further indicate curcumin and resveratrol can interact synergistically to enhance antioxidant efficacy, anti-inflammatory potential, and anticancer properties (11–14). However, the utility of these bioactive nutrients is often constrained by poor water solubility, low bioavailability, and chemical instability, limiting their widespread application (15). Consequently, there is an imperative need to develop robust delivery systems that can enhance the bioavailability of curcumin and resveratrol.

The construction of nano-scale colloidal delivery systems has emerged as an effective strategy to overcome the technical limitations (16). Among the various options, delivery carriers fabricated from natural biomacromolecules (e.g., protein-polysaccharide complexes) have garnered considerable attention due to their exceptional biocompatibility, controllable degradability, and high encapsulation efficiency (17, 18). For instance, fucoidan-coated zein-based nanocomposites have developed for resveratrol encapsulation (19), and zein-alginate oligosaccharide nanocomposites for curcumin loading (20). However, most colloidal systems have been designed to encapsulate either curcumin or resveratrol individually. The simultaneous co-encapsulation of both compounds poses significant challenges due to their differential molecular polarity (21). Although co-encapsulation of both bioactive compounds has been achieved by preparing two separate delivery carriers followed by physical mixing, this approach is complicated and often result in substantial bioactive components leakage. Consequently, highly efficient co-delivery systems for curcumin and resveratrol have rarely been reported.

**Figure 1:**
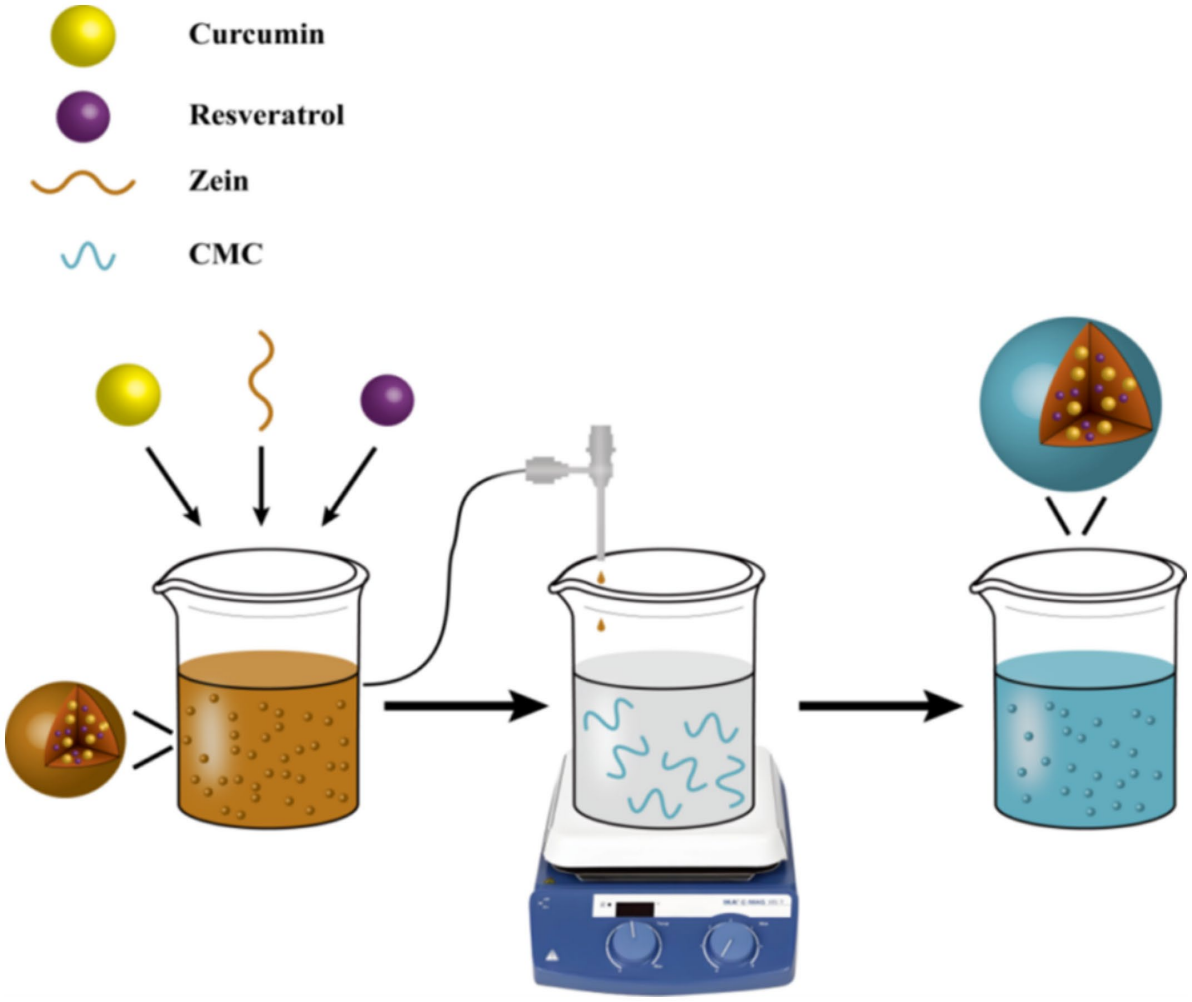
Schematic diagram of formation of CRZC NPs.

In this study, we developed a protein-polysaccharide self-assembled system for dual-polyphenol co-delivery, achieving synergistic encapsulation of curcumin and resveratrol through molecular structure design. As natural bio-based nanomaterials, zein and carboxymethyl chitosan (CMCS) exhibit superior biocompatibility and biodegradable properties, effectively evading the metabolic clearance limitations associated with synthetic nanomaterials. Hydrophobic functional components (curcumin and resveratrol) were encapsulated within zein’s hydrophobic core, followed by directional assembly of CMCS through electrostatic interactions between its anionic groups and the positive charges on zein surface, yielding unique zein-CMCS nanocomposites (Figure 1). The effect of zein: CMCS mass ratio (4:1 to 1:2) on nanocomplex characteristics (mean size, zeta-potential, and encapsulation efficiency) of zein-CMCS nanocomplexes was investigated. Furthermore, we evaluated the encapsulated system’s effects on the *in vitro* antioxidant activity and bioaccessibility of the co-encapsulated nutraceuticals using a simulated gastrointestinal model. This work establishes design principles for colloidal co-delivery systems targeting multiple bioactive compounds.

## 2 Materials and methods

### 2.1 Materials

Zein (97% purity, AR grade) from corn was purchased from Macklin Co. Ltd. (Shanghai, China). CMCS, with a deacetylation degree of 95% and a carboxylation degree of 70%, was obtained from Jinke Pharmaceutical (Zhejiang, China). Curcumin (98% purity) and resveratrol (99% purity) was purchased from Sigma-Aldrich (USA). All other reagents are analytical grade and used as received.

### 2.2 Preparation of curcumin/resveratrol-loaded zein/CMCS nanoparticle

The curcumin/resveratrol-loaded zein NPs (CRZ NPs) was prepared by the anti-solvent method with slightly modifications (22). Curcumin (500 mg), resveratrol (500 mg) and zein (10.0 g) were dissolved in 1.0 L of 70% ethanol solution and magnetically stirred for 1 h to prepare a transparent solution. Secondly, the solution was injected into 3.0 L of distilled water at 600 rpm stirring, and then the CRZ NPs were spontaneously formed. Thirdly, CMCS (0.5% w/v) powder was dispersed in distilled water by continuous stirring for 6 h at 20°C. Fourthly, the CRZ NPs solution was injected into the CMCS solution, and the curcumin/resveratrol-loaded zein/CMCS nanoparticles (CRZC NPs) were formed. The resulting was evaporated at 40°C in a rotary evaporator to remove the ethanol.

### 2.3 Encapsulation efficiency and loading capacity

The EE and LC of the curcumin and resveratrol in the NPs was determined in accordance with previously described methods (15). Briefly, freshly prepared dispersions were centrifuged at 12,000 r/min for 30 min and the NPs were separated. The obtained supernatant was diluted with ethanol solution, and the concentrations of curcumin and resveratrol were determined by analyzed using a UV spectrophotometry (UV 2600, Shimadzu Co., Tokyo, Japan) at 426 nm and 306 nm, respectively. Suitable calibration curves were prepared to calculate the curcumin and resveratrol concentrations. The EE and LC was determined by the following Equation:


EE=(Encapsulated curcumin)/(Total curcumin)×100%

$$\mathrm{LC}=(\text{Encapsulated curcumin})/(\begin{array}{l} \text{Total mass of}\;\\ \text{nanocomplexes} \end{array})\times 100\%$$

EE=(Encapsulated resveratrol)/(Total resveratrol)×100%

$$\mathrm{LC}=(\text{Encapsulated resveratrol})/(\begin{array}{l} \text{Total mass of}\;\\ \text{nanocomplexes} \end{array})\times 100\%$$

### 2.4 Characterization of NPs

The mean particle size, polydispersity (PDI), and zeta-potential (*ξ*) of the NPs were measured with a dynamic light scattering (DLS) instrument (Anton Paar Litesizer 500, Austria). Before analysis, the samples were diluted 5 times to minimize multiple scattering effect. These measurements were performed in quadruplicate. The morphology of the freeze-dried NPs was determined using an SEM (SU8010, Hitachi, Japan) at an accelerating voltage of 15.0 kV. Before observation, the sample was coated with a gold layer under a sputter coater. The Fourier transform infrared spectroscopy (FT-IR) spectra of the samples were measured using a Thermo Nicolette 6,700 spectrophotometer (Thermo Fisher Scientific Co., Ltd., MA, United States) from 400 cm^−1^ to 4,000 cm^−1^ at a resolution of 4 cm^−1^. The X-ray diffraction (XRD) patterns of the samples were obtained by a Bruker AXS D8 Advance X-ray diffractometer (Bruker Inc., Germany) equipped with Ni-filtered Cu Kα radiation. The 2θ angle range used was from 5 to 60°.

### 2.5 Simulated gastrointestinal digestion

According to Zhang’s method (23), the release profiles of curcumin and resveratrol from NPs were quantified under simulated gastrointestinal conditions: simulated gastric fluid (SGF) (3.2 mg/mL pepsin, 2.0 mg/mL NaCl in HCl, pH 2.0) and simulated intestinal fluid (SIF) (2.0 mg/mL pancreatin, 12.0 mg/mL bile salts, 8.8 mg/mL NaCl, 6.8 g/L K₂HPO₄, pH 7.4) at 37°C. Fresh NP dispersions (30 mL) were mixed with 30 mL SGF and incubated at 37°C. Aliquots (2 mL) were collected at 30, 60, and 90 min, with equal volumes of fresh SGF replenished. After gastric digestion, the entire digest was transferred to 60 mL SIF. Intestinal phase samples (2 mL) were withdrawn at 120, 150, 180, 210, and 240 min, replacing with fresh SIF. All samples were centrifuged (6,000 × g, 5 min) to pellet undigested material. Supernatants were diluted and analyzed for released curcumin/resveratrol content via UV–Vis spectrophotometry (Shimadzu UV-2600, Tokyo, Japan) as described in Section 2.3. The *in vitro* bioaccessibility of the polyphenols was then calculated using the following equation:


$$\text{Bioaccessibility}\;(\%)=(\begin{array}{l} \text{Concentration of polyphenol in}\;\\ \text{the micelle phase}/\text{Concentration}\;\\ \text{of polyphenol in the overall}\;\\ \text{digesta}\;\mathrm{at}\;\text{the}\;\mathrm{end}\;\text{of digestion} \end{array})\times 100$$

### 2.6 Antioxidant activity evaluation

The DPPH and ABTS radical scavenging activity was analyzed according to the previous studies (24, 25), with slight modifications. In brief, 2 mL of the sample was mixed with 2 mL of the DPPH solution (0.1 mM), and the resulting mixture was placed in the dark and allowed to react for 30 min. The absorbance at 517 nm was recorded by using a UV–Vis spectrophotometer (UV 2600, Shimadzu Co., Tokyo, Japan). In the same way, ABTS solution (7.4 mM) was mixed with a potassium persulfate solution (2.6 mM) at a 1:1 v/v ratio and the mixture was then placed in the dark for 12 h. Exactly 3.9 mL of diluted ABTS· + solutions, which had an absorbance of about 0.7 at 734 nm, were mixed with 0.1 mL of water or samples for 5 min and then measured at 734 nm. The DPPH and ABTS radical scavenging activity was calculated using Equation:


Scavenging activity(%)=((Ab−As)/As)×100%

where Ab and As were the absorbance values of control solution and samples, respectively.

### 2.7 Storage stability

The CRZ, and CRZC NPs dispersions were stored under refrigerated conditions (4°C) for one month. The retention rate (%) of the two nutraceuticals after long-term storage were measured using the method described in Section 2.3 and calculated using the following equations:


$$\begin{array}{l} \text{Retention}\;\\ \text{rate of}\;\mathrm{Cur}\;(\%)=(\begin{array}{l} \text{the amount of}\;\\ \mathrm{Cur}\;\text{after storage} \end{array})/(\begin{array}{l} \text{the initial}\;\\ \text{amount of}\;\mathrm{Cur} \end{array})\times 100\% \end{array}$$

$$\begin{array}{l} \text{Retention}\;\\ \text{rate of}\;\mathrm{Res}\;(\%)=(\begin{array}{l} \text{the amount of}\;\\ \mathrm{Res}\;\text{after storage} \end{array})/(\begin{array}{l} \text{the initial}\;\\ \text{amount of}\;\mathrm{Res} \end{array})\times 100\% \end{array}$$

### 2.8 Statistical analysis

The values were presented as the mean ± standard deviation (SD) of each treatment. The data were analyzed using analysis of variance (ANOVA) in the SPSS 20.0 software package (IBM, New York). The significant was defined at the 95% confidence level.

## 3 Results and discussion

### 3.1 Particle size and zeta-potential analysis

The CRZC NPs were successfully fabricated via anti-solvent precipitation (Figure 1). Co-dissolution of zein with curcumin/resveratrol in 70% ethanol enabled hydrophobic interaction-driven embedding of polyphenols within zein’s hydrophobic domains. Ethanol-to-water phase diffusion triggered zein self-assembly, forming primary nutraceutical-loaded cores. Subsequent interfacial assembly with CMCS via electrostatic and hydrogen bonding yielded nanostructures, consistent with Qin et al.’s protein-polysaccharide interaction models (26).

**Figure 2:**
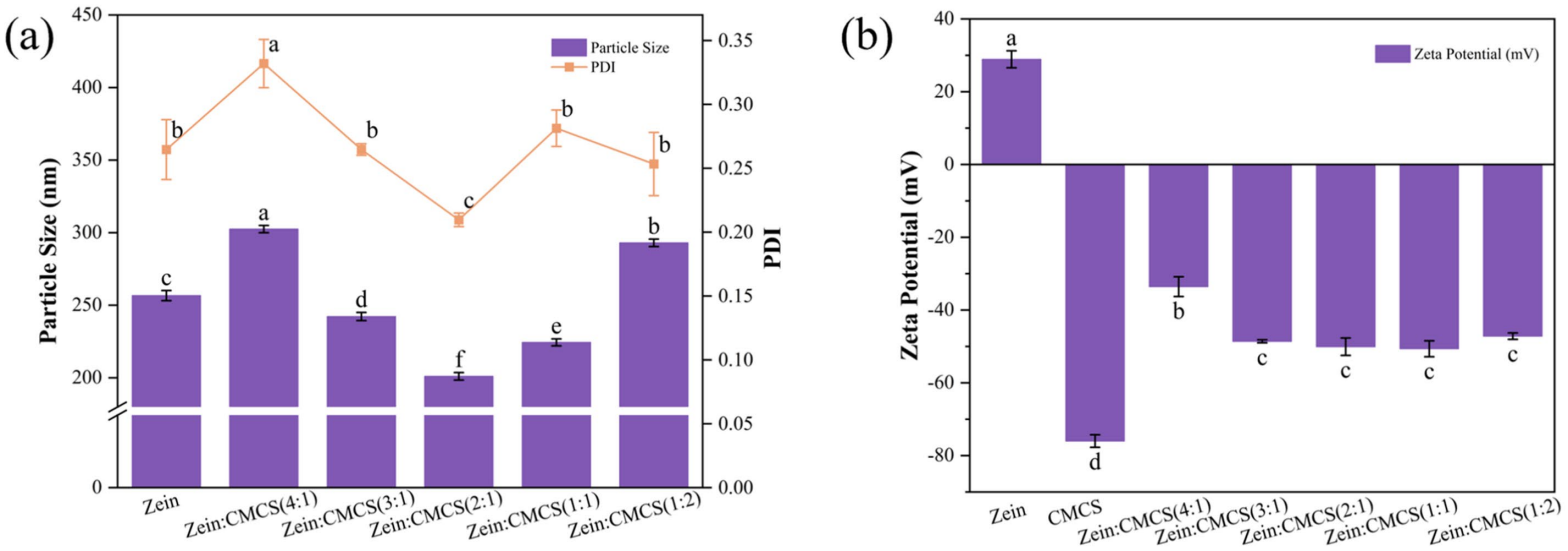
Effect of zein: CMCS mass ratio on the mean particle size and PDI **(a)** and zeta-potential **(b)** of NPs. Different lowercase letters signified significant differences.

Particle size analysis (Figure 2) revealed that pristine zein NPs exhibited an average hydrodynamic diameter of 256.5 ± 3.4 nm (PDI = 0.26), consistent with anti-solvent precipitation efficacy for hydrophobic proteins nanoassembly. This size regulation stems from rapid ethanol diffusion –induced polarity changes that trigger zein conformational rearrangement and subunit aggregation (27). Notably, the curcumin/resveratrol-loaded system (CRZ NPs) showed a 11.7% reduction in mean particle size (226.5 ± 2.5 nm) compared to pristine zein NPs, indicating polyphenol incorporation promotes denser nanostructures via modulated zein interactions (15). This observation aligns with the mean particle size reduction pattern reported by Yang et al. for co-delivery of quercetin and resveratrol in zein/carboxymethyl cellulose NPs (24).

The introduction of CMCS induced significant size modulation of CRZ NPs. At a zein: CMCS mass ratio of 4:1, the composite system displayed an increased average diameter of 302 ± 2.8 nm, primarily due to NPs aggregation caused by insufficient CMCS concentration. Conversely, optimal size minimization (201 ± 2.5 nm) with superior dispersion stability (PDI = 0.21) was achieved at a 2:1 mass ratio (zein: CMCS). This indicates that moderate CMCS coating suppresses aggregation via steric hindrance, whereas excessive polysaccharide layers increase hydrodynamic diameter. These findings consistency with Lin et al., confirming the threshold effect of polyelectrolyte concentration on NPs stability (22).

Zeta potential analysis (Figure 2b) demonstrated that pristine zein NPs exhibited a positive charge of +28.9 mV at pH 4.0 (pI = 5.9). The CRZ NPs exhibited a slightly reduced surface potential (+28.5 mV), indicating partial surface distribution of bioactive components. Upon CMCS coating, all composites transitioned to strongly negative potentials (−34.2 to −41.6 mV), confirming the successful formation of stabilizing shells through anionic polysaccharide adsorption. This charge inversion is consistent with observations by Huang et al.’s (28), who noted negative zeta potential shifts in zein/pectin nanocomposites, illustrating successful surface deposition of anionic pectin. Similarly, Li et al. (20) reported comparable charge inversion patterns in zein-alginate oligosaccharide systems. These insights underscore the pivotal role of CMCS in modulating nanoparticle characteristics, potentially paving the way for advanced applications in nutrition and bioactive delivery systems.

### 3.2 EE and LC analysis

**Figure 3:**
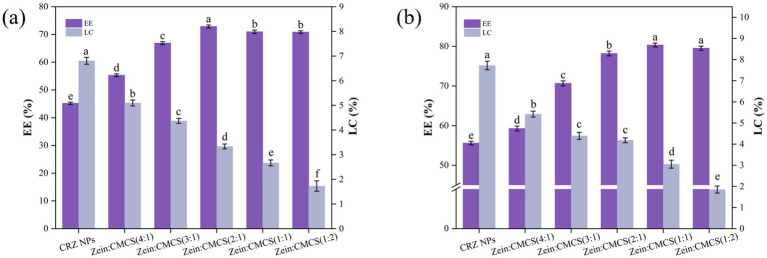
Effect of zein: CMCS mass ratio on EE and LC of NPs, **(a)** curcumin and **(b)** resveratrol. Different lowercase letters signified significant differences.

Figure 3 showed the EE and LC of curcumin and resveratrol in both CRZ and CRZC NPs. In uncoated CRZ NPs, the EE of curcumin and resveratrol reached only about 45.2 and 55.6%, respectively, indicating incomplete encapsulation or potential leakage post-formulation. Notably, resveratrol exhibited higher encapsulation across all nanoformulation compared to curcumin, likely due to its higher compatibility with the hydrophobic core of zein nanoparticles. These findings are consistent with previous results obtained using the anti-solvent precipitation method for co-encapsulating curcumin and resveratrol co-encapsulating zein matrix (10, 29, 30).

The incorporation of CMCS enhanced the EE of curcumin and resveratrol in CMCS-coated CRZ NPs, confirming the anionic polysaccharide coating improves the retention of bioactive compound. CMCS increased of the system’s hydrophilicity, prompting more substantial encapsulation of hydrophobic curcumin/resveratrol within the zein NPs (22). Moreover, some of the curcumin and resveratrol may reside within the polysaccharide shell formed by the CMCS (24). Other studies support these findings: when curcumin and resveratrol were co-encapsulation within zein-epigallocatechin gallate conjugates using an anti-solvent precipitation method followed by rhamnolipid biosurfactant coating, encapsulation efficiencies were 71% for curcumin and 85% for resveratrol (29). Similarly, zein-ethyl cellulose core-shell NPs yielded encapsulation efficiency of 54% for curcumin and 71% for resveratrol, respectively (30). These results align with other research indicating that hydrophilic polyelectrolyte coatings enhance EE in zein-based nanocarriers (15, 31).

Further studies revealed that the concentration CMCS critically modulates loading characteristics: At a zein: CMCS mass ratio of 2:1, optimal EE (72.90%) was achieved, while LC decreased as the CMCS proportion increased. This inverse correlation arises because excessive polysaccharide coating increasing carrier mass, reducing practical LC efficacy post-saturation, a finding consistent with Yang et al. (24). Their study on zein/carboxymethyl cellulose NPs for co-encapsulating quercetin-resveratrol similarly found an increase in EE with higher carboxymethyl cellulose mass, though the opposite was true for LC.

Thus, considering factors such as small particle size, high EE and LC, CRZC NPs with a zein to CMCS mass ratio of 2:1 was selected for further experimentation.

### 3.3 FT-IR and XRD analysis

**Figure 4:**
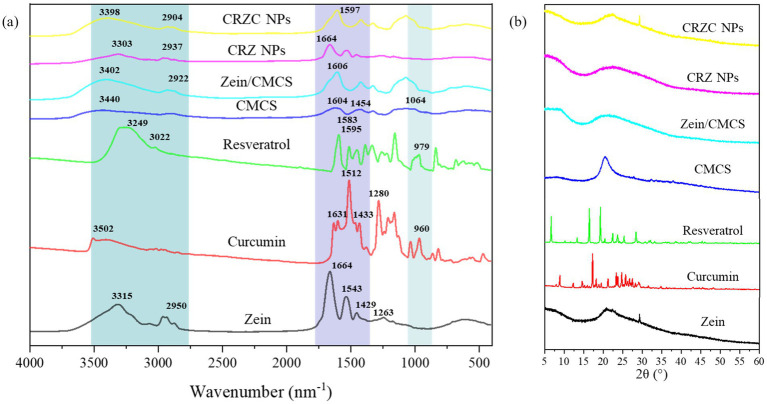
FTIR spectra **(a)** and XRD spectra **(b)** of samples.

To investigate the intermolecular interactions within NPs, FT-IR spectroscopy was conducted on curcumin, resveratrol, zein, CMCS and NPs. Figure 4a illustrated that all the samples exhibited a broad band between 3,100–3,500 cm^−1^, indicating the presence of O-H stretching vibrations. Native resveratrol exhibited characteristic bands at 979 cm^−1^ for trans-olefinic band, 1,595 cm^−1^ for C=C aromatic stretching (32). For curcumin, peaks at 1631 cm^−1^, 1,512 cm^−1^, 1,433 cm^−1^ and 1,280 cm^−1^ corresponded to aromatic ring and benzene ring vibrations, including C=C, C=O, C-O and C-C stretching (33). Zein displayed absorption bands at 1664 cm^−1^, 1,543 cm^−1^, 1,429 cm^−1^, and 1,263 cm^−1^, attributed to hydrophobic C-H, amide I (-C=O), amide II (-C-N and -N-H), and amide III (34). Similarly, CMCS exhibited peak at 1604 cm^−1^, 1,583 cm^−1^, 1,454 cm^−1^, and 1,064 cm^−1^, corresponding to C=O, CH_2_COO-, and C-O-C group vibrations, respectively (35). Upon integration of curcumin and resveratrol with zein, the O-H peak shifted from 3,320 cm^−1^ to 3,336 cm^−1^, along with amide I peak shifted from 1,664 cm^−1^ to 1,649 cm^−1^. These shifts suggest the involvement of hydrophobic effects and hydrogen bonding in the formation of the CRZ NPs. Coating the CRZ NPs with CMCS did not cause significant spectral changes, indicating that no covalent bonds formed between zein and CMCS. Notably, the characteristic peaks of curcumin and resveratrol were absent in both CRZ NPs and CRZC NPs, indicating complete encapsulation within the NPs. The shifts observed in the CRZC NPs, particularly around 3,200–3,400 cm^−1^ (O-H and N-H) and 1,597 cm^−1^ and 1,431 cm^−1^ (amide II), indicated the presence of electrostatic interactions and hydrogen bonds between zein NPs and CMCS.

The crystalline diffraction patterns of the samples were shown in Figure 4b. The native curcumin exhibited multiple distinct characteristic peaks at 2θ of 8.8°, 12.4°, 17.3°, 18.1°, 24.7°, confirming its crystalline nature. Similarly, native resveratrol displayed distinct crystalline peaks at 2θ angles of 13.3°, 16.4°, 19.3°,22.4°, 23.6°, 25.3°, and 28.3°. Zein had a broad t peak at diffraction angles of 20.1°, and CMCS had only a single broad peak at 20.2°, reflecting their amorphous properties as a protein and polysaccharide, respectively. The characteristic peaks of curcumin and resveratrol were not found in the NPs, likely due to their low concentration in the NPs. Additionally, the peak intensities of CRZC NPs at 20° were significantly reduced compared to those of pure zein and CMCS. This reduction suggests the formation of new noncovalent interaction between zein and CMCS, corroborated by the FT-IR results. Similar findings have been observed in previous studies involving the encapsulation of curcumin (36) or resveratrol (37) in a polymers matrix.

### 3.4 Microstructure analysis

**Figure 5:**
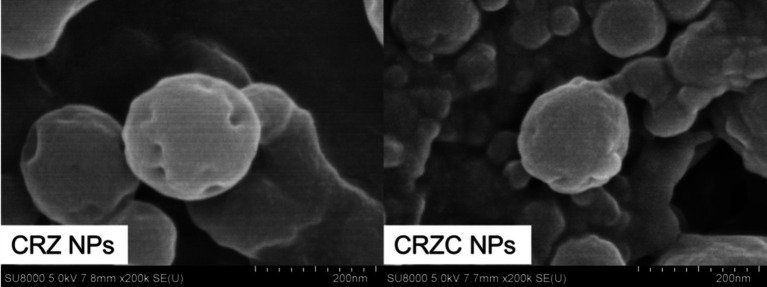
SEM images of samples.

Scanning electron microscopy (SEM) revealed distinct morphological features of NPs (Figure 5). CRZ NPs exhibit spherical morphology with smooth surfaces, consistent with intrinsic zein self-assembly during anti-solvent precipitation (15). CMCS-coated CRZC NPs maintained spherical morphology but demonstrated reduced particle size compared to CRZ NPs, corroborating the result of dynamic light scattering (DLS) analysis. The composite system showed a gel-like matrix interspersed among NPs, attributable to excess CMCS. Critically, no free curcumin or resveratrol crystals was observed, confirming complete polyphenols encapsulation. These findings correlate well with FT-IR and XRD analyses.

### 3.5 Antioxidant activity evaluation

**Figure 6:**
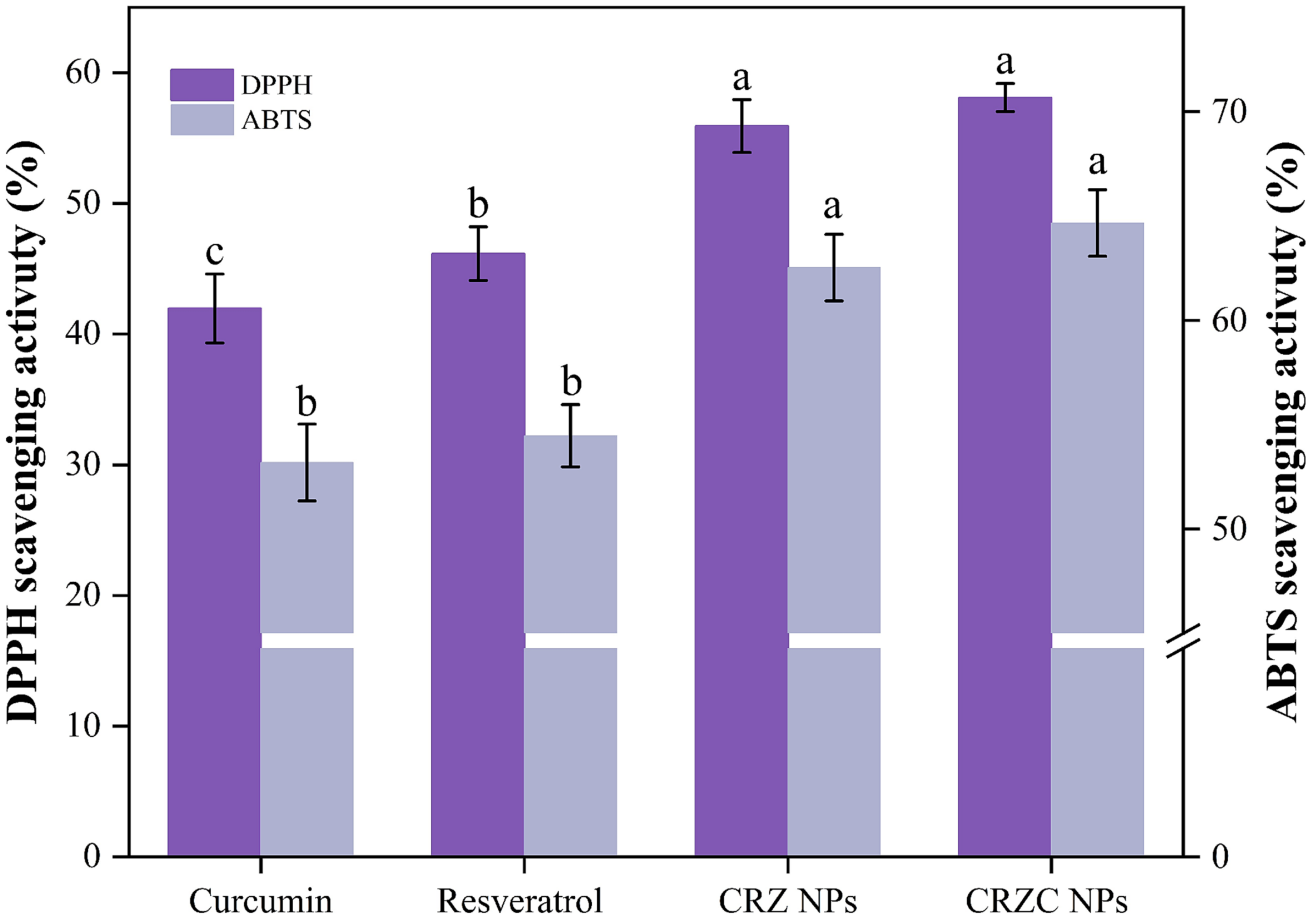
DPPH and ABTS+ radical scavenging ability of free curcumin, resveratrol, CRZ NPs, and CRZC NPs. Different lowercase letters signified significant differences.

The antioxidant efficacy of free versus nanoencapsulated curcumin/resveratrol was systematically evaluated using DPPH and ABTS radical scavenging assays (Figure 6). Nanoencapsulation significantly enhanced radical scavenging capacity: CRZC NPs exhibited 58.10% higher DPPH scavenging than free curcumin (*p* < 0.05), while ABTS scavenging increased by 64.67% (*p* < 0.05). Resveratrol-containing systems showed analogous enhancement trends. These results demonstrate that zein/CMCS-based nanocarriers not only preserve the intrinsic antioxidant activity of encapsulated compounds but also enhance radical scavenging efficiency compared to free components. This suggests synergistic interactions between carrier materials (zein and CMCS) and bioactive constituents (29). These findings indicate that combinatorial loading of functional components can amplify the antioxidant potential of zein NPs, underscoring their promise for functional applications.

### 3.6 Release profiles of polyphenol under simulated gastrointestinal digestion

**Figure 7:**
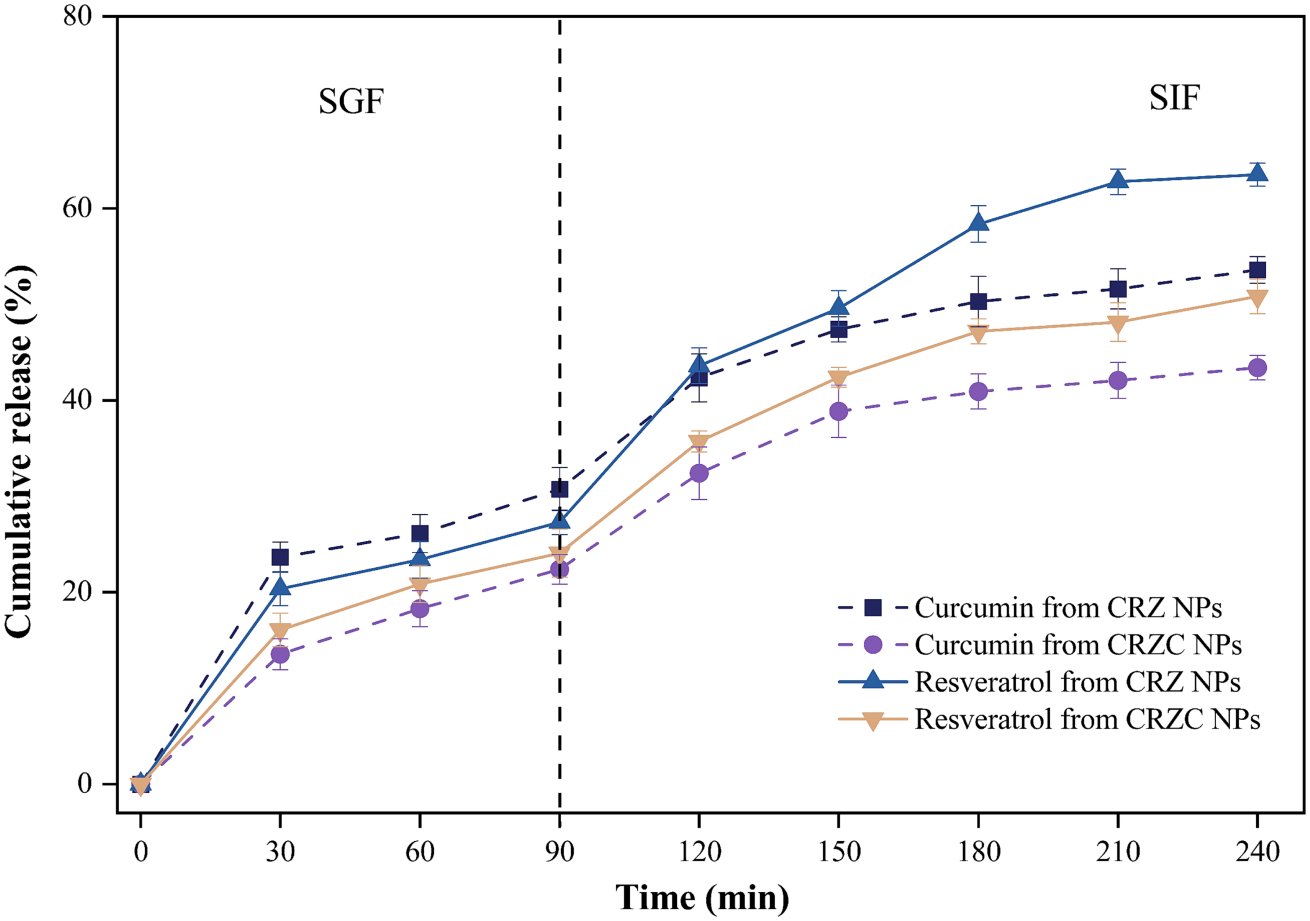
Release profile of curcumin (dotted lines) and resveratrol (solid lines) from NPs under simulated gastrointestinal conditions.

Release profile of co-encapsulated curcumin and resveratrol from NPs were quantified during sequential simulated gastric (SGF) and intestinal (SIF) phases, as depicted in Figure 7. CRZ and CRZC NPs exhibited sustained-release characteristics for both curcumin and resveratrol. Notably, curcumin displays lower solubilization in SGF compared to resveratrol, attributed to resveratrol’s higher solubility. This observed behavior aligns with previous reports on similar co-delivery nanocarriers of these compounds (30). During the SIF phase, both CRZ and CRZC NPs exhibited burst release effects, potentially due to the influence of bile salts and peptides present in the SIF, which facilitate the dissolution of bioactive compounds. Additionally, the release rate from CRZ NPs surpassed that from CRZC NPs, possibly because the CMCS layer acts as a physical barrier, limiting protease access to the zein, and concurrently enhancing the diffusion of the encapsulated compounds into the surrounding medium, thereby controlling curcumin release (38, 39). Furthermore, resveratrol exhibited a higher retention rate than curcumin, likely because resveratrol is more easily accommodated within the hydrophobic interiors of the zein nanoparticles, which may stem from differences in molecular interaction properties (19).

**Figure 8:**
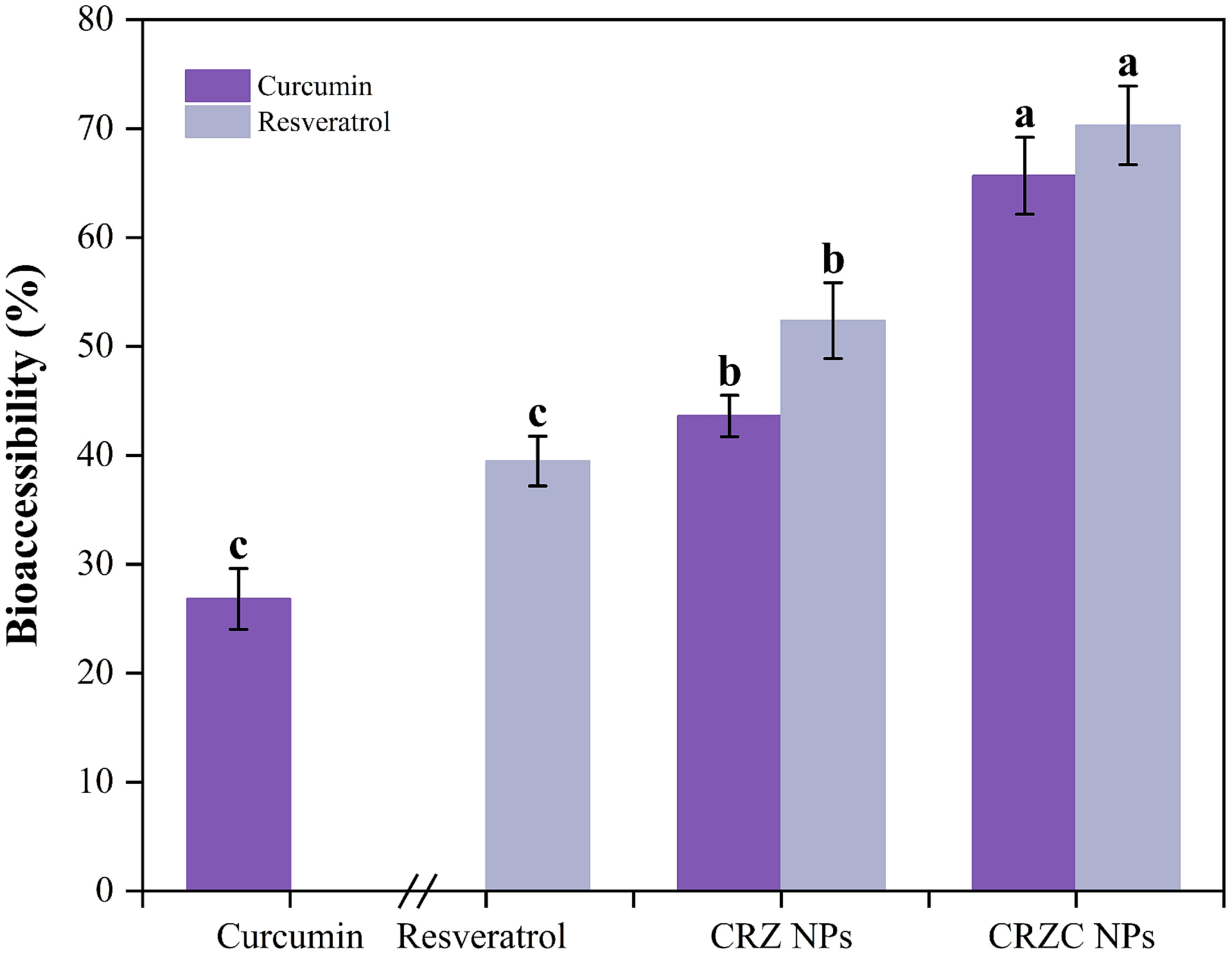
Bioaccessibility of curcumin and resveratrol from NPs. Different lowercase letters signified significant differences.

Bioaccessibility analysis (Figure 8) revealed free curcumin and resveratrol at 26.82 and 39.48%, respectively, limited by precipitation in aqueous media. Nanoencapsulation improved bioaccessibility (curcumin: 43.62%; resveratrol: 52.37%), with CMCS-coated NPs outperforming uncoated systems (*p* < 0.05). This enhancement stems from: (i) sustained release minimizing precipitation, (ii) enhanced micellar solubilization through CMCS-bile salt synergism, and (iii) reduced enzymatic degradation. The differential bioaccessibility between compounds (resveratrol>curcumin) aligns with their solubility profiles in intestinal conditions. These findings align with NPs studies demonstrating prevention of polyphenol aggregation (40), confirming CRZC NPs as promising carriers for targeted intestinal delivery of hydrophobic nutraceuticals.

### 3.7 Storage stability

**Figure 9:**
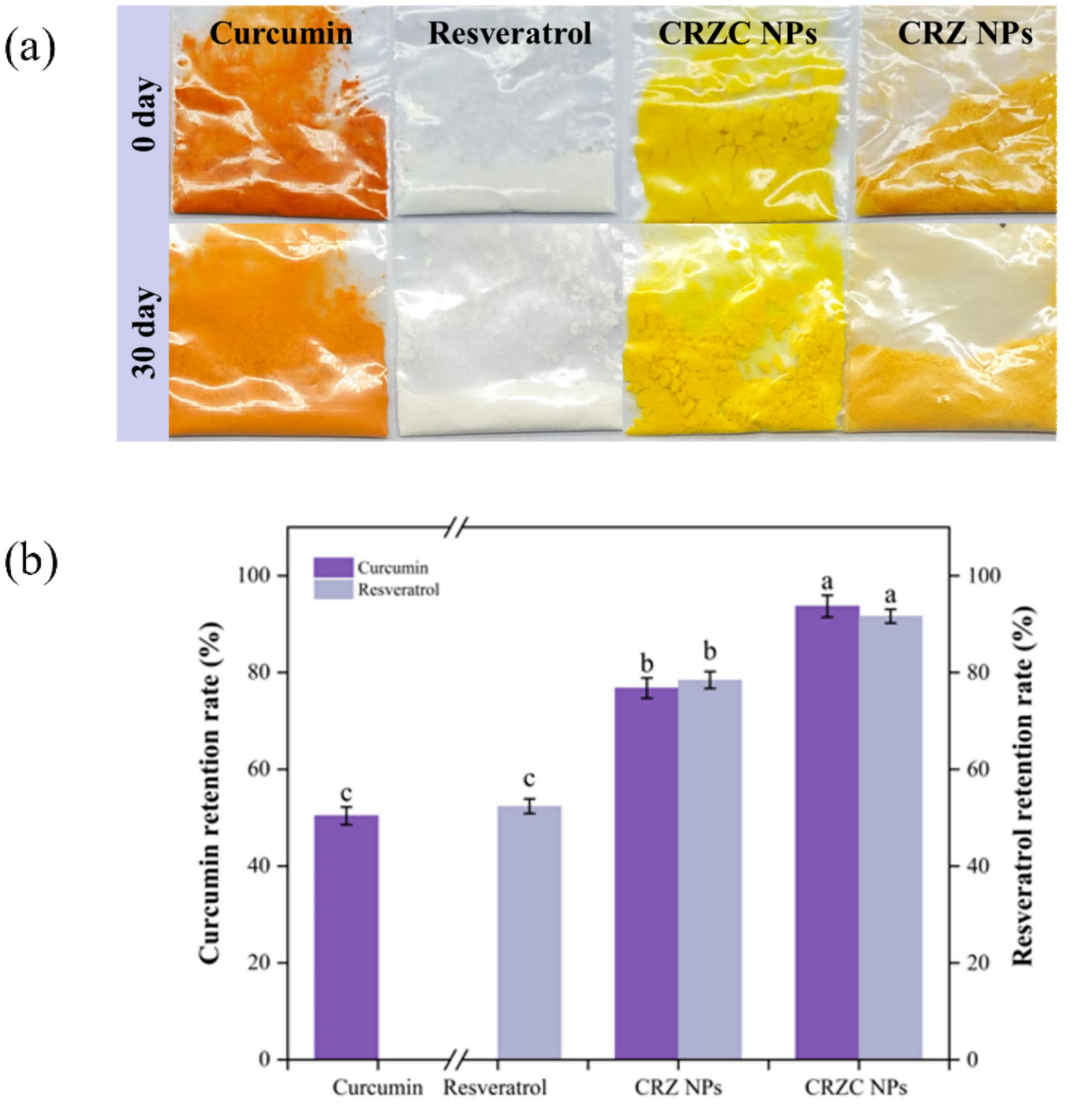
The retention rates of curcumin and resveratrol in CRZ NPs and CRZC NPs after storage. Storage appearance **(a)**, retention rate **(b)**. Different lowercase letters signified significant differences.

Long-term storage stability of CRZ and CRZC nanoparticles was evaluated over 30 days (Figure 9). CRZ nanoparticles experienced notable degradation; curcumin retention dropped to 76.76, and resveratrol to 78.43 (*p* < 0.05 relative to initial values). This degradation was attributed to colloidal aggregation and oxidation of polyphenols on the surface. In contrast, CRZC nanoparticles showed markedly better performance (*p* < 0.05) with significantly higher retention of both active compounds compared to CRZ nanoparticles. This improvement underscores the effectiveness of the core-shell architecture, where the CMCS coating provides electrostatic barriers that prevent particle coalescence, decrease oxygen permeability by densifying the matrix, and shield polyphenols from aqueous radicals. Therefore, the core-shell structure formed by CMCS and zein substantially enhances the physicochemical stability of nanocarriers, offering long-term protection for curcumin and resveratrol (15).

## 4 Conclusion

This study successfully constructed a composite nanodelivery system based on zein and CMCS, achieving synergistic co-encapsulation of curcumin and resveratrol. Structural characterization confirmed that the polyphenolic bioactive compounds primarily localized within the hydrophobic core regions of zein, while CMCS formed an electrostatically stabilized protective shell. Compositionally optimized CRZC NPs exhibit superior encapsulation performance for both hydrophobic bioactive components. Notably, the nanoencapsulated bioactive components retained effective free radical scavenging capacity, indicating the encapsulation process had minimal impact on their functional groups. The CMCS coated CRZC NPs also improved storage stability and enabled controlled release kinetics in the gastrointestinal environment, thereby enhancing bioaccessibility. These findings establish zein/CMCS NPs as a promising platform for functional food applications requiring targeted delivery of hydrophobic nutraceuticals.

## Data Availability

The raw data supporting the conclusions of this article will be made available by the authors, without undue reservation.
